# Development and Validation of a Novel Hypoxia Score for Predicting Prognosis and Immune Microenvironment in Rectal Cancer

**DOI:** 10.3389/fsurg.2022.881554

**Published:** 2022-04-25

**Authors:** Kaiyan Yang, Zhaolong Shen, Ning Yin, Jun Quan, Mengwen Wang, Kai Gao

**Affiliations:** Department of Gastrointestinal Surgery, The Third Xiangya Hospital of Central South University, Changsha, China

**Keywords:** hypoxia, neutrophils, activated memory CD4 + T cells, metastasis, rectal cancer

## Abstract

Hypoxia plays a major role in various tumor types. However, few studies have concentrated on the prognostic model of hypoxia-related genes in rectal cancer and the effect of hypoxia on neutrophil-mediated immunosuppression. We performed Kaplan–Meier analysis, random survival forest analysis, and Cox regression analysis on 342 hypoxia-related genes, constructed hypoxia score in the Gene Expression Omnibus (GEO) cohort, and verified them in the Cancer Genome Atlas (TCGA) cohort. Then the patients were divided into two groups according to the risk level. The overall survival rate of the high-risk (HRisk) group was significantly higher than that of the low-risk (LRisk) group (GEO, *p* < 0.001; TCGA, *p* = 0.016). Through receiver operating characteristic and decision curve analysis, the nomogram based on hypoxia score has excellent prediction ability. Functional enrichment analysis showed that hypoxia, metastasis, inflammation, immunity, and other related pathways were enriched. The HRisk group was associated with lower tumor purity, higher immune and stromal score, higher neutrophils, and lower activated memory CD4 + T cells. More importantly, the checkpoint of neutrophil-mediated immunosuppression increased in the HRisk group. In conclusion, a hypoxia score based on 5 hypoxia-related genes can be used to predict the prognosis of rectal cancer and ANLN with a cancer-suppressing effect and SRPX (Sushi Repeat Containing Protein X-Linked) with a cancer-promoting effect may be potential therapeutic targets for rectal cancer.

## Introduction

Rectal cancer is a type of colorectal malignancy, accounting for two-thirds of colorectal cancer, and its incidence rate is increasing year by year, and tends to be younger (1, 2). Currently, rectal cancer is treated by mainly surgical resection (3), and the 5-year survival rate is ~60% (4). The invasion and metastasis of rectal cancer are important causes of prognosis and death (5). Therefore, it is necessary to further study the molecular mechanism of invasion and metastasis and develop more accurate antitumor therapy.

Hypoxia is a universal sign of solid tumor growth microenvironment. Previous studies have confirmed that hypoxia is related to cancer extracellular matrix, angiogenesis, and cancer cell metastasis and plays a central regulatory role in tumor invasion and metastasis (6, 7). In addition, some recent studies have confirmed the direct relationship between hypoxia and tumor microenvironment. In immunotherapy, in the face of anoxic environment, T lymphocytes will increase the expression of CD137 (8); under hypoxic conditions, stabilizing Nrf1 impairs the polarization of tumor-associated macrophages. In addition, hypoxia drives CD8 + T effector function and cell migration (9), suggesting that hypoxia can activate different responses of immune cells and tumor cells. Although there have been many studies on the changes in the immune microenvironment caused by hypoxia, few studies have paid attention to the effect of hypoxia on neutrophils.

In the course of this study, through a systematic review and patient transcriptome data and corresponding clinical data finished in Gene Expression Omnibus (GEO) and The Cancer Genome Atlas (TCGA) databases, we comprehensively collected the genes related to hypoxia. First, we used these genes to establish a polygenic feature in the GEO cohort, and then used this feature to pre-match the patients. Finally, we tested and verified them in the TCGA cohort. More importantly, to research the possible mechanism, we studied the relationship through functional enrichment analysis between hypoxia and neutrophil elevation and the decrease in activated memory CD4 + T cells. We also further explored the role of each gene in the changes of tumor-immune microenvironment induced by hypoxia.

## Materials and Methods

### Data Collection and Collation

In the present study, 190 rectal cancer samples originated from GEO (GSE87211) (Supplementary Table S1). And we also get the data from TCGA database (Supplementary Table S2). The gene expression profile, survival information, and clinical characteristics of rectal cancer patients were downloaded, and the data were collected and applied according to TCGA guidelines. The inclusion criteria are as follows: all patients must have completed follow-up information and RNA-seq data. By following the gene annotation package, the gene ID of the corresponding data set is converted into the corresponding gene symbol. The analysis excluded RNA that could not be detected in more than 10% of the samples.

### Construction and Validation of a Hypoxia-Related Prognostic Signature

We used the “LIMMA (version 3.50.1)” R software package to analyze the differential expression between tumor samples and adjacent non-tumor samples in the GEO cohort. By univariate Cox analysis, the prognostic hypoxia-related genes were determined. To reduce the number of genes required for prognostic markers, random survival forest analysis was performed using the “randomForestSRC” package. Then we rank the importance of these genes, select the five most important genes (variable importance > 0.005) for combined analysis, and try to establish a multivariate Cox regression model. Risk scores were calculated based on the mRNA expression of each gene and its corresponding multivariate Cox regression coefficient. Patients were divided into high-risk (HRisk) groups and low-risk (LRisk) groups according to the median of each combined risk score, and Kaplan–Meier analysis was performed. Patient risk scores were calculated based on the same genes and coefficients trained in the GEO cohort, and patients were then stratified into two groups in the TCGA cohort as a validation cohort.

### Survival Analysis and Assessment of the Prognostic Signature

Kaplan–Meier analysis and Cox regression analysis were used to test the difference in overall survival (OS) between the HRisk and LRisk groups. “timeROC” R package was used for time-dependent receiver operating characteristic curve analysis to assess the predictive efficiency of prognostic signals. On this basis, the independent predictors obtained by multivariate Cox regression were integrated, and the prediction nomogram was constructed with “reglot” and “RMS” R software package. The prediction ability was evaluated by a corresponding calibration chart. Then, the clinical benefit difference of the combined nomogram was investigated by decision curve analysis (DCA).

### Functional Enrichment Analysis

The analysis of Gene Ontology (GO) and Kyoto Encyclopedia of genes and genes (KEGG) was carried out by the “clusterProfiler” R package based on all differentially expressed genes (DEGs) (10). In addition, to estimate the activation degree of 50 HALLMARK pathways, the “MSigDB” R package (version 1.2.0) and the “GSVA” R package (version 1.14.1) were applied under standard setting. Based on the GSVA score, the “LIMMA” R software package was used to analyze the difference HALLMARK path between the two groups of HRisk patients in the two cohorts.

### Tumor-Immune Microenvironment Analysis

We compared the immune score, interstitial score, immune cell infiltration, and immune-related genes between the HRisk and LRisk groups to describe the characteristics of tumor-immune microenvironment for prognostic markers. The immune score and stromal score were quantified by the “estimate” R package. We estimated the abundance of immune cells using “cibersort” and “xcell” algorithms (11). In addition, we calculated the correlation between 22 immune cells and the risk score of prognostic markers. The “limma” R software package was used to analyze the differential expression of immune-related genes between the HRisk and LRisk groups. The “gsva” R software package was then used to calculate the correlation between the signature gene and 22 immune cells. In the Human Protein Atlas (HPA) database, the single-cell type map shows the expression of protein-coding genes in single-cell types and the number of genes detected in cell types. The expression levels of the hub gene of mRNA and protein in different cell types were detected by the tool. To validate the prognostic DEG, gene expression and prognostic data were downloaded from the GSE17536 dataset using the PrognoScan online tool. To further confirm the reliability of prognostic DEG, we examined antibot-based protein expression data from HPA in normal and tumor tissues.

### Statistical Analysis

The OS probability of the HRisk and LRisk groups was analyzed by the Kaplan–Meier method and univariate and multivariate Cox regression analyses. We use Fisher's exact test to determine the difference in the proportion of somatic mutations, and false discovery rate (FDR) < 0.01 was considered to be statistically significant. We used the Mann–Whitney U test to compare the immune score, stromal score, and immune cell infiltration between the two groups, and the value of *p* was adjusted by the BH method. We used Spearman's correlation analysis to estimate interactions. A qualified significance level was considered to be 0.05, and all statistical values of *p* were two-sided if not specifically mentioned in the text. Statistical analysis was performed using R version 4.1.2.

## Results

### Construction of Hypoxia Score

**Figure 1:**
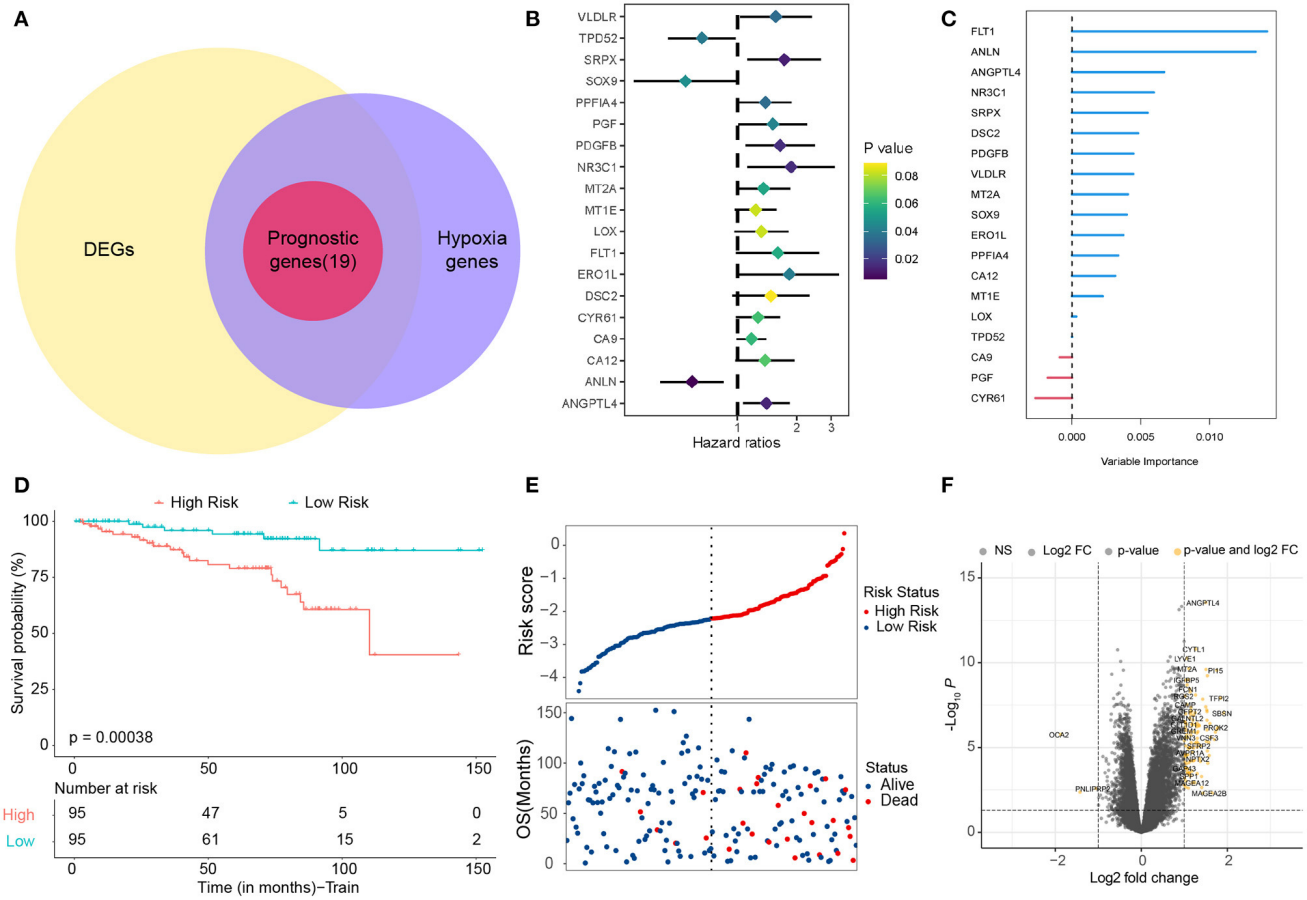
Identification of hypoxia-related candidate genes. **(A)** Venn diagram to identify prognostic hypoxia-related genes. **(B)** Forest plots showing the results of the univariate Cox regression analysis. **(C)** Random survival forest analysis screened 5 genes sorted by importance. **(D)** Kaplan–Meier curves for the OS of patients between the high-risk and low-risk groups. **(E)** The distribution of the risk scores and the distributions of OS status. **(F)** Volcano map shows differentially expressed genes. OS, overall survival.

Based on previous studies (12, 13), we found 342 hypoxia-related genes (Supplementary Table S3). In total, 83.6% (286 / 342) of hypoxia-related genes were differentially expressed in tumor tissue and adjacent tissue. It is shown by univariate Cox regression analysis that 19 (5.6%) of hypoxia-related genes were prognostic genes of OS (Figure 1B) and overlapped between prognostic genes and the DEGs (Figure 1A). We used a random survival forest analysis to assign importance to each hypoxia-related prognostic DEG to reduce the number of genes in prognostic molecules (Figure 1C). The first five genes of importance ranking were screened out. On the basis of multivariate Cox regression, the prognostic scores of five genes were constructed according to the proportional risk (PH): hypoxia score = (0.21^*^expression level of FLT1) + (−0.75^*^expression level of ANLN) + (0.32^*^expression level of ANGPTL4) + (−0.21^*^expression level of NR3C1) + [0.37^*^expression level of SRPX (Sushi Repeat Containing Protein X-Linked)]. Patients were divided into HRisk groups (*n* = 95) and LRisk groups (*n* = 95) according to the median risk score (Figure 1D). Mortality was higher in the HRisk group than in the LRisk group (Figure 1E). The differential genes of HRisk and LRisk are shown in Figure 1F.

### Construction and Validation of Nomogram Model

**Figure 2:**
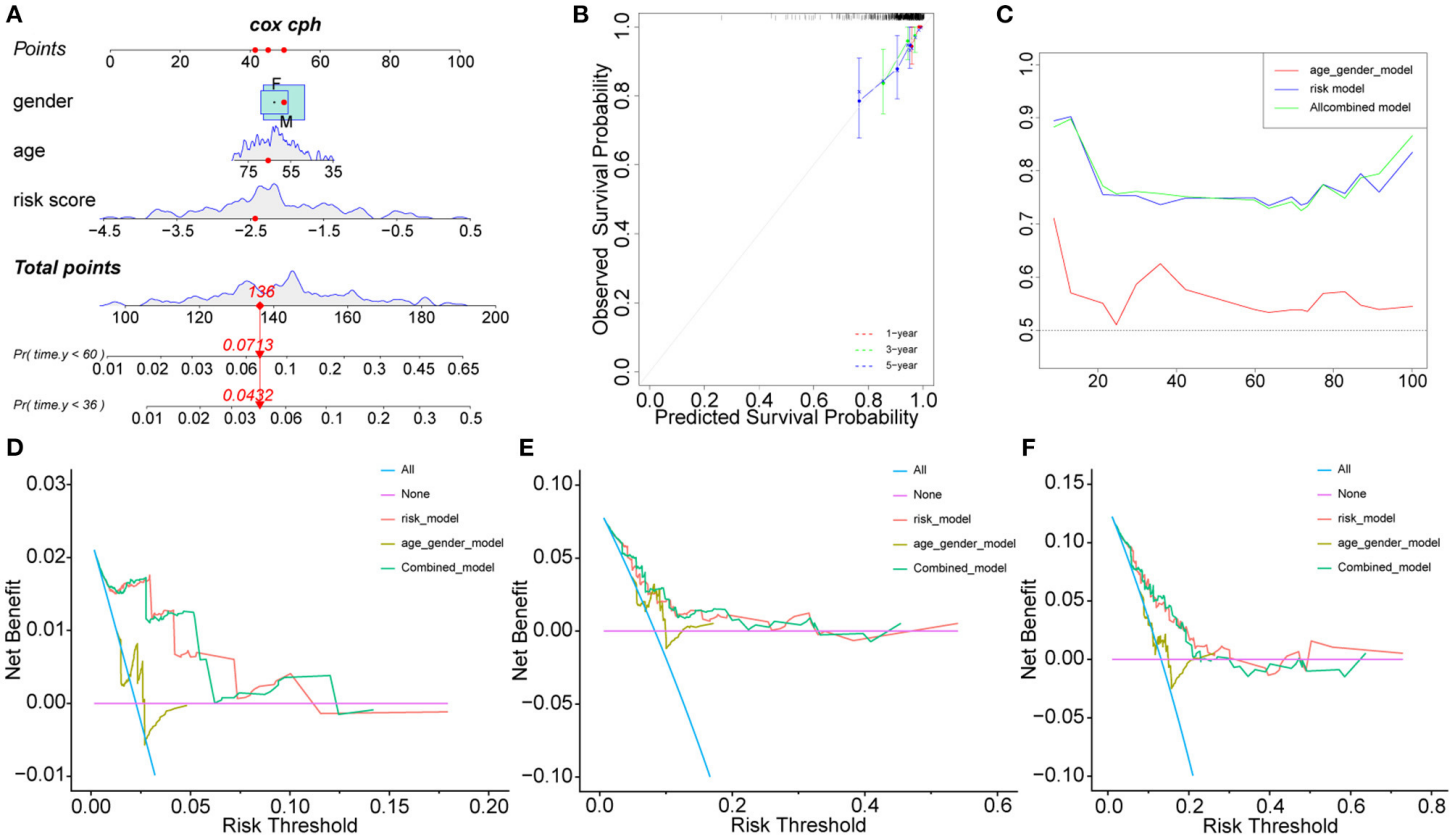
Nomogram model construction in GEO cohort. The development of a nomogram to predict the 3-, and 5-year OS **(A)** and the corresponding calibration **(B)**. **(C)** Area under the curve of time-dependent receiver operating characteristic (ROC) curves. **(D–F)** Decision curve analyses of the nomograms based on OS for 1-, 3-, and 5-year risk. OS, overall survival.

The clinical characteristics of the two cohorts are summarized in Supplementary Tables S4, S5. In addition to risk scores, gender and age were also considered independent predictors of rectal cancer (14, 15). To further improve the prediction ability, a nomogram based on clinical factors and risk scores was established in the GEO cohort (Figure 2A). The calibration curves of nomograms showed great consistency (Figure 2B). The Areas under the Curve (AUCs) of 1, 3, and 5 years in the GEO cohort were 0.903, 0.734, and 0.746, respectively (Figure 2C), indicating that nomograms have good stability and prediction performance. In addition, DCA assessed the predictive value of nomograms for clinical decision-making at 1, 3, and 5 years in both cohorts (Figures 2D–F). The results show that the curve of nomogram is higher than the curve of risk score or clinical factors, but it is very close to the curve of risk score, indicating that the risk score and nomogram have high reliability. In addition, we used the TCGA cohort to verify the risk score and nomogram. We found that in the TCGA cohort, there was a significant difference between HRisk and LRisk (Supplementary Figure S1A). The nomogram calibration curve combined with clinical factors showed excellent consistency (Supplementary Figure S1B). The AUCs of 1, 3, and 5 years were > 0.7 (Supplementary Figure S1C). DCA showed that the nomogram had good clinical decision-making significance (Supplementary Figures S1D–F).

### Potential Functional Analyses of the Hypoxia Score

**Figure 3:**
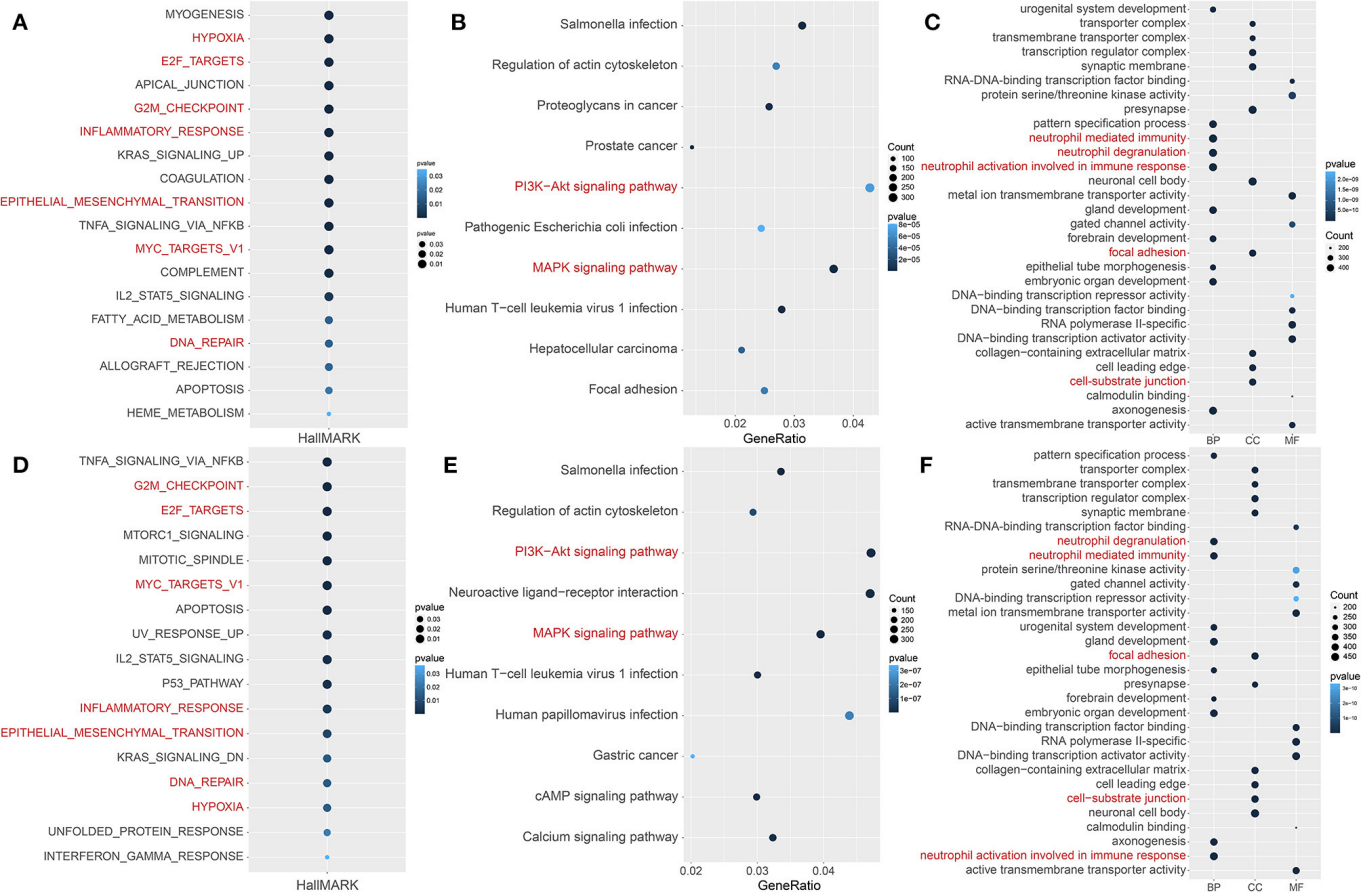
Potential functional analyses in the GEO cohort. **(A)** GSVA analysis of hallmark pathways between the HRisk and LRisk groups was performed. **(B,C)** KEGG and GO pathway analyses of differentially expressed genes between HRisk and LRisk groups. **(D)** GSVA analysis of hallmark pathways between cluster 2 and cluster 3 groups was performed. **(E,F)** KEGG and GO pathway analyses of differentially expressed genes between cluster 2 and cluster 3 groups. GO, Gene Ontology; KEGG, Kyoto Encyclopedia of genes and genes; HRisk, high-risk; LRisk, low-risk.

To further analyze functions related to hypoxia, the GSVA scores of 50 landmark pathways between the HRisk and LRisk groups were analyzed. We found that it was mainly related to hypoxia, inflammatory response, metastasis (epithelial–mesenchymal transition), and proliferation (E2F targets and G2M checkpoint) (Figure 3A). The “cluster profile” R package was used for KEGG and GO enrichment analysis according to the DEG between the HRisk and LRisk groups in the GEO queue. As shown in Figure 3B, the KEGG analysis indicated that these DEGs were enriched in PI3K-Akt and MAPK signaling pathways. The overlapping GO functional pathways between the two groups are mostly rich in the pathways related to neutrophil-mediated immune response and metastasis-related pathways (Figure 3C). To further test the prediction of risk scores on the degree of hypoxia, we used 342 hypoxia genes in unsupervised cluster GEO patients and obtained three subtypes (Supplementary Figures S2A–C). There were significant prognostic differences between cluster 2 and cluster 3 (Supplementary Figure S2D). Further using GSVA difference analysis and KEGG and GO enrichment analyses of cluster 2 and cluster 3, we also found that hypoxia, inflammatory response, metastasis, immunity, and other related pathways were enriched (Figures 3D–F). More importantly, we used the TCGA cohort to verify the enrichment pathway between risk scores, HRisk and LRisk. Interestingly, inflammatory response, metastasis, and immunity-related pathways are also enriched (Supplementary Figures S3A–C).

### Correlation Between Hypoxia and Tumor-Immune Microenvironment

**Figure 4:**
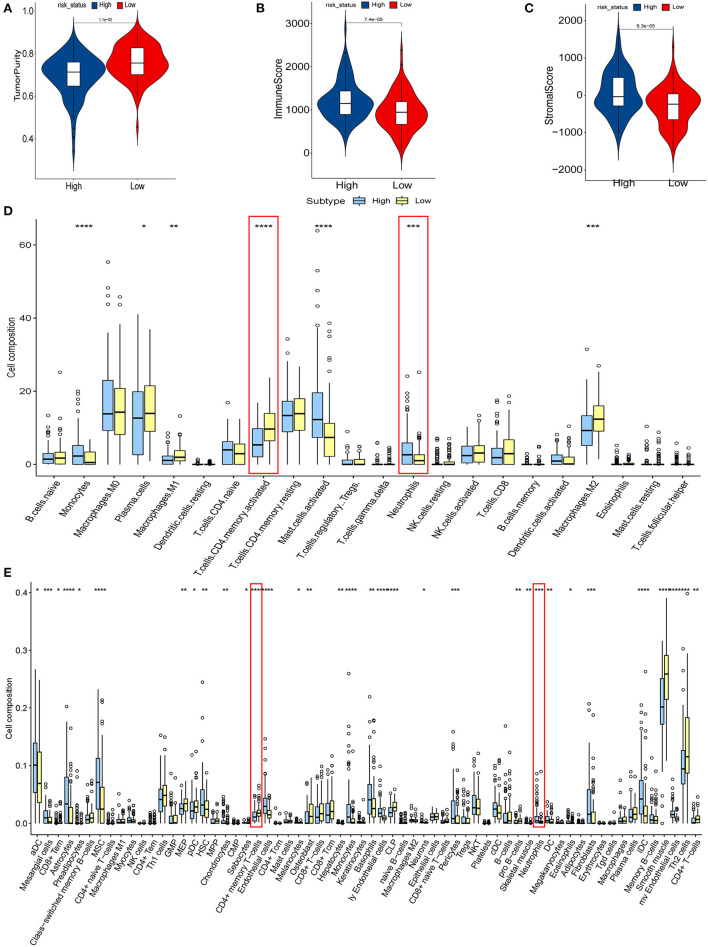
The relationship between hypoxia and tumor-immune microenvironment in the GEO cohort. Comparison of TumorPurity **(A)**, ImmuneScore **(B)**, and StromalScore **(C)** between the HRisk and LRisk patients. **(D)** Boxplots depicting the CIBERSORT scores of 22 immune cells of the HRisk patients compared to the LRisk patients. **(E)** Boxplots depicting the Xcell scores of 64 immune cells of the HRisk patients compared to the LRisk patients. HRisk, high-risk; LRisk, low-risk. *P < 0.05, **P < 0.01, ***P < 0.001, ****P < 0.0001.

We explored the relationship between hypoxia and tumor-immune microenvironment to further explain the difference in survival between the two groups. It was observed that LRisk patients were related to higher tumor purity in both cohorts, while HRisk patients were related to higher immune scores and higher stromal scores (Figures 4A–C, Supplementary Figures S4A–C). Interestingly, in the GEO cohorts, HRisk patients were related to higher neutrophils and lower activated memory CD4+ T cells according to two different immune cell infiltration algorithms, Cibersort and Xcell (Figures 4D,E). More importantly, we got consistent results in the TCGA queue. Neutrophils increased significantly in HRisk patients and activated memory CD4+ T cells decreased in HRisk patients (Supplementary Figures S4D,E).

**Figure 5:**
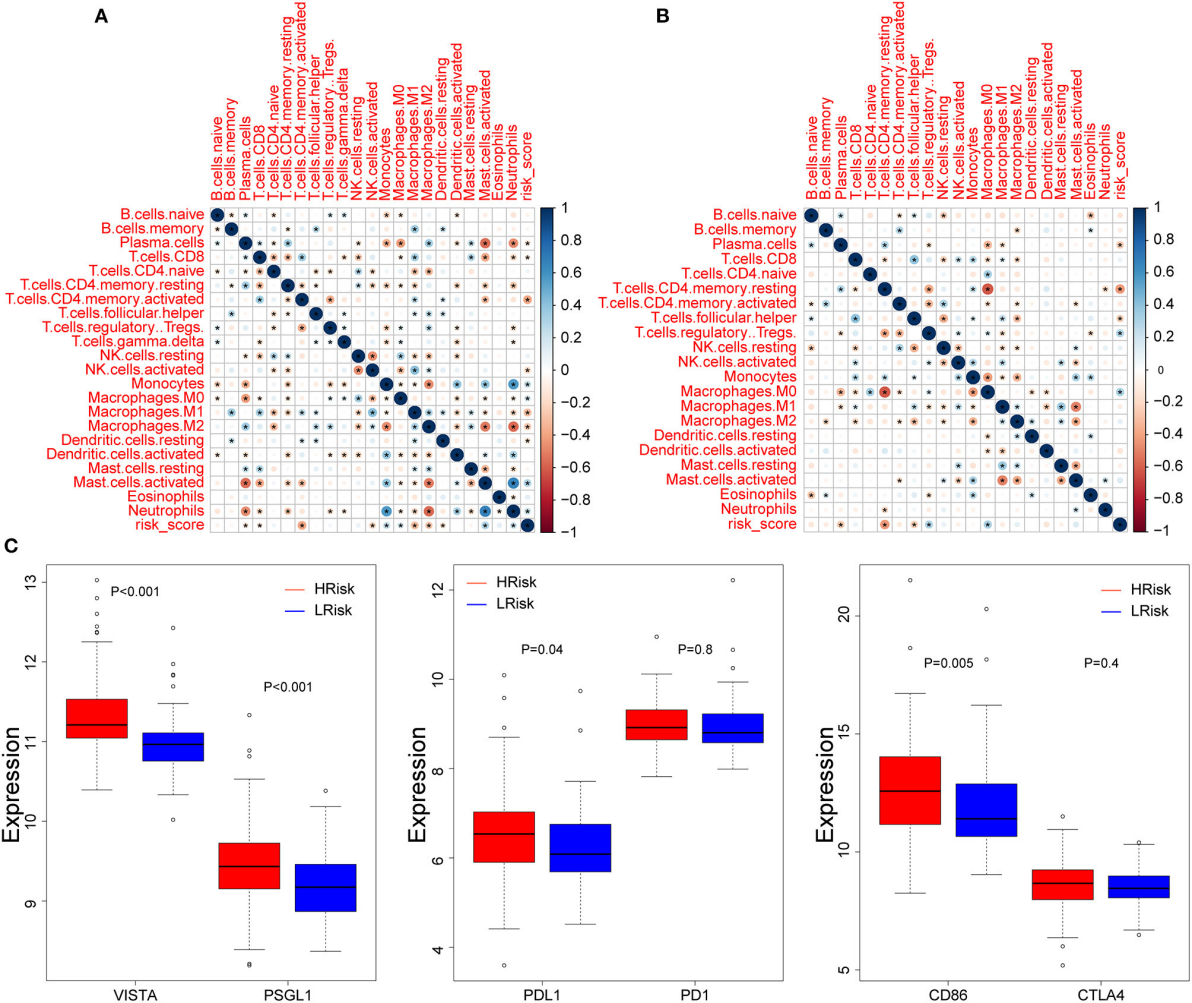
Correlation between immune infiltration and risk score. Correlation between 22 CIBERSORT cell types and risk score in the GEO **(A)** and TCGA **(B)** cohorts. **(C)** Boxplots depicting the expression of neutrophil-mediated T cell immunosuppressive targets between the high-risk and low-risk groups in the GEO cohort. *P < 0.05.

**Figure 6:**
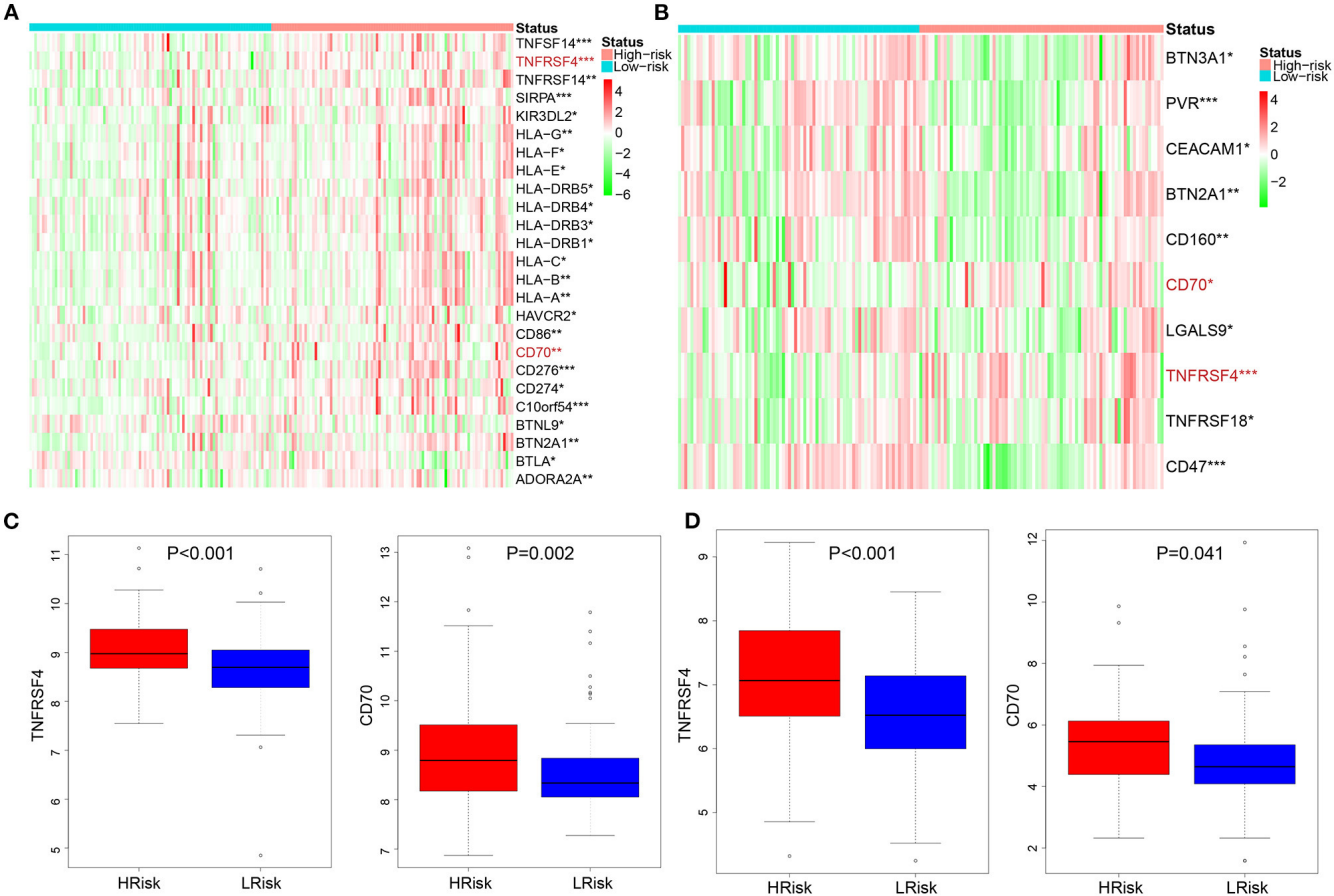
Analysis of immune checkpoint between the HRisk and LRisk groups. Heatmap depicting the differences in immune checkpoint between the HRisk and LRisk patients in the GEO **(A)** and TCGA **(B)** cohorts. **(C,D)** The compassion of two representative immune checkpoints in two cohorts. HRisk, high-risk; LRisk, low-risk. *P < 0.05, **P < 0.01, ***P < 0.001.

We calculated the correlation between risk score and infiltrated immune cells to further explore the relationship between hypoxia and immune cell infiltration. We found that in the GEO cohort, the risk score was positively correlated with neutrophils (COR = 0.22), and negatively correlated with activated memory CD4+ T cells (COR = −0.39) (Figure 5A); in the TCGA database, neutrophils were positively correlated with risk score (COR = 0.01), while activated memory CD4 + T cells were negatively correlated with risk score (COR = −0.11) (Figure 5B). To further probe into the relationship between neutrophils and T cells, we detected neutrophil-mediated immunosuppressive targets and relative receptors (Figure 5C). We found that ligands (VISTA, PDL1, CD86) expressed by neutrophils were highly expressed in HRisk, T cell surface receptor PSGL1 was significantly highly expressed in the HRisk group, and PD1 and CTLA4 were also highly expressed in HRisk, although there was no statistical significance. Immune microenvironment plays an essential role in the malignant progression of tumors. Therefore, we explored the relationship of immune checkpoints (16) between HRisk and LRisk groups (Supplementary Table S4). We found that 31.6% (25/79) of checkpoints in the GEO queue were meaningful (Figure 6A) and 12.7% (10/79) of checkpoints in the TCGA queue were meaningful (Figure 6B). More importantly, HRisk was associated with higher TNFRSF4 and CD70 in GEO (Figure 6C) and TCGA (Figure 6D) cohorts.

### Identification of Key Genes Based on Hypoxia Score

**Figure 7:**
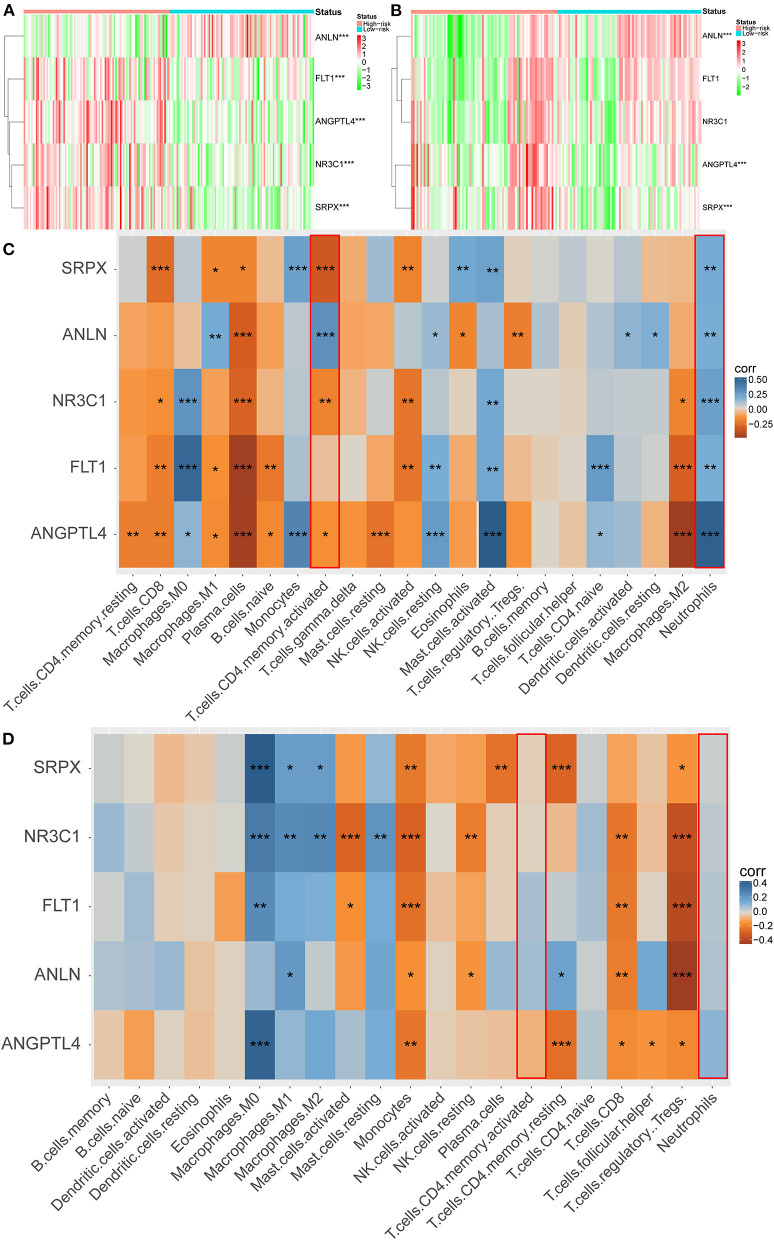
Correlation between immune infiltration and 5 genes. Heatmap plot for expression of 5 genes between the high-risk and low-risk groups in the GEO **(A)** and TCGA **(B)** cohorts. **(C,D)** Heatmap depicting the correlation between 5 genes with the ssGSEA scores of 22 immune cells. *P < 0.05, **P < 0.01, ***P < 0.001.

**Figure 8:**
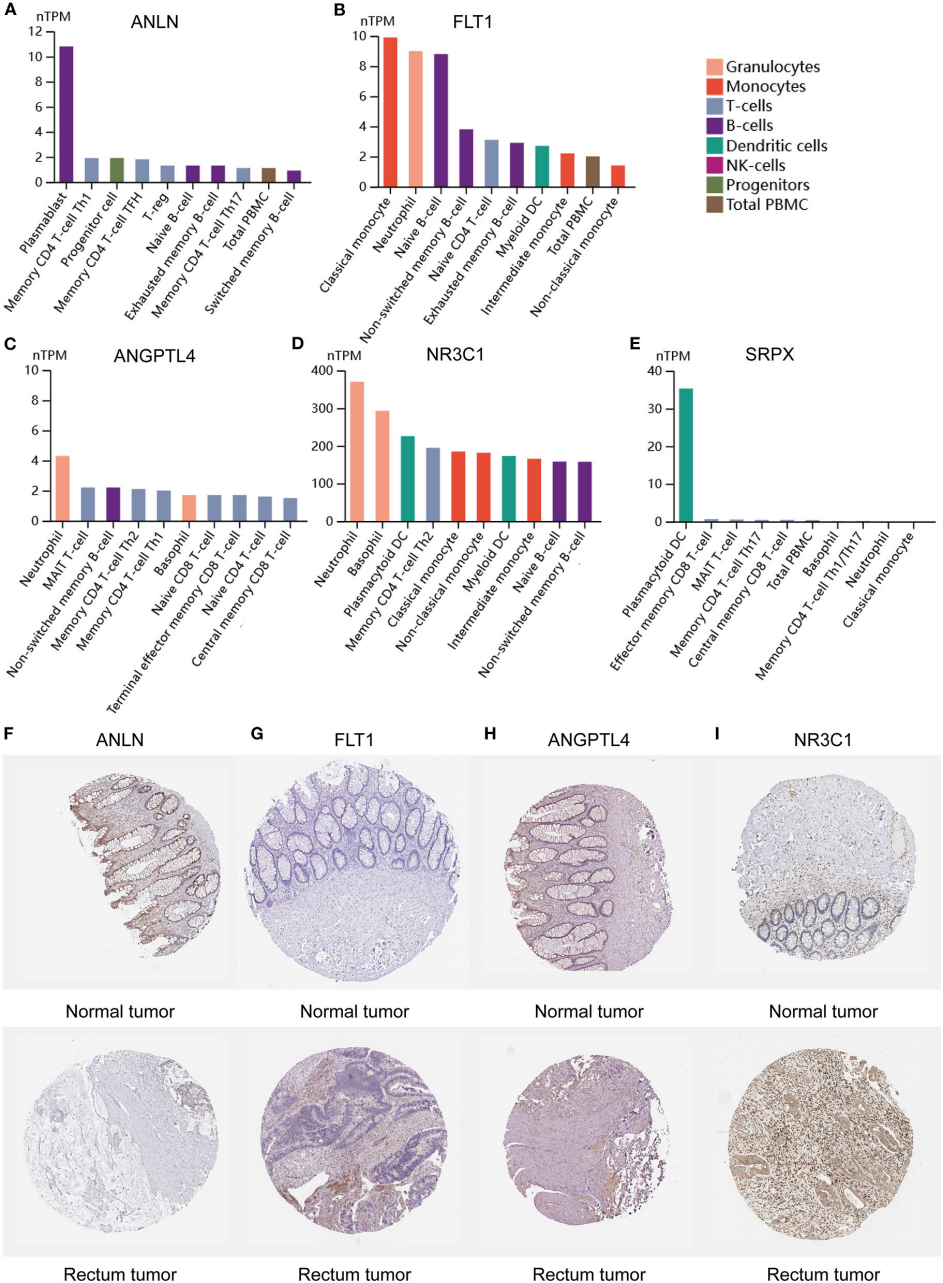
Expression of 5 genes in different immune cell lines and tissues. **(A–E)** The expression of hub genes in cell types. **(F–I)** Immunohistochemical staining for the key genes in normal tissues and rectum cancer tissues.

**Figure 9:**
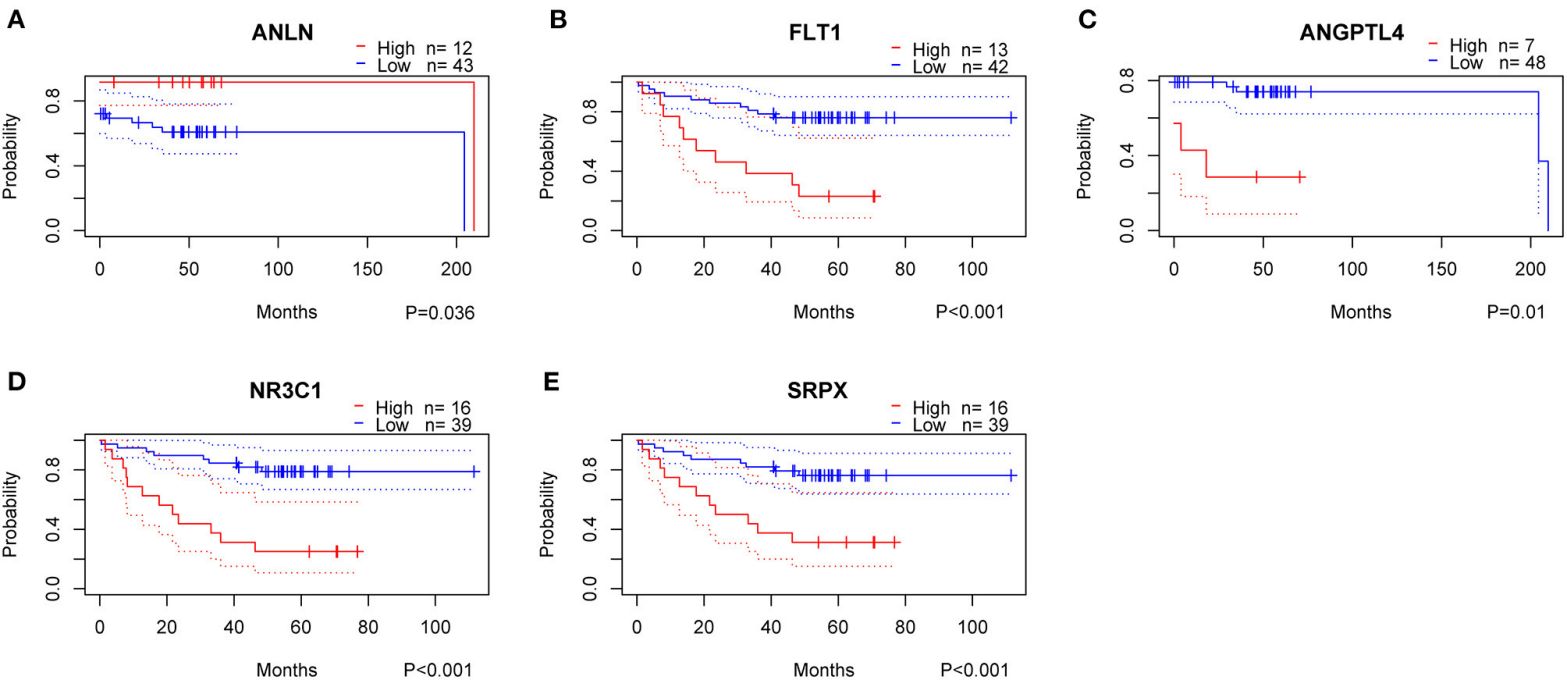
Validation of prognostic genes using data from the GEO database. High, High Expression; Low, Low Expression.

To clarify the role of the five genes in the risk score, we explored their expression between HRisk and LRisk. The results showed that ANLN was expressed at a higher level in the LRisk group, while ANGPTL4 and SRPX were expressed at higher levels in the HRisk group (Figures 7A,B). In addition, after performing an analysis of the correlation between 5 genes and single-sample gene set enrichment analysis (ssGSEA) scores of 22 immune cells, five genes were positively correlated with neutrophils, and activated memory CD4+ T cells are positively correlated with ANLN and negatively correlated with four other genes (Figures 7C,D). In addition, the expression levels of five genes in different immune cells were detected in HPA. We found that the expression levels of ANLN and SRPX were very low in neutrophils, whereas FLT1, ANGPTL4, and NR3C1 were highly expressed in neutrophils (Figures 8A–E). In order to verify the reliability of five genes in predicting prognosis, by using HPA to detect protein expression levels in normal and tumor tissues, we found that there were significant differences in ANLN and NR3C1 between tumor tissues and normal tissues (Figures 8F–I). To further verify the prognostic significance of five genes, we used the PrognoScan online tool to download the GSE17537 data set for verification. ANLN is associated with a good prognosis, and the other four genes are associated with a poor prognosis (Figures 9A–E). These genes may have some guiding significance in the prognosis and treatment of rectal cancer.

## Discussion

Due to the heterogeneity of tumors, even after comprehensive treatment, patients with rectal cancer still have a significant risk of recurrence and metastasis. Consequently, developing reliable molecular biomarkers that inform clinical practice and improve clinical outcomes is an urgent task. We identified a 5-gene hypoxia-related signature in the GEO cohort and validated it in the TCGA cohort.

Hypoxia is the term of hypoxia supply, which is equivalent to 8–10 mmHg, which will damage the biological work of cells (17). Under hypoxia, hypoxia-inducible factors migrate to the nucleus and mediate multiple gene regulation involving angiogenesis, energy metabolism, apoptosis, and metastasis (18–20). Although these findings confirm the important link of hypoxia in cancer development and prognosis, the link between hypoxia-related genes and OS of patients with rectal cancer is still a huge unknown. Different from the previous LASSO method, we adopt a new algorithm to screen as few genes as possible to create prognostic markers. Our risk scores provide accurate predictions of prognosis for patients with rectal cancer, and HRisk patients have shorter survival time. Nomograms that combine with important clinical factors can provide guidance for improving the accuracy of clinical decision-making and carrying out stratified treatments. DCA showed that nomogram had better predictive potential than risk score and clinical factors alone. These results suggest that the risk score is a biomarker for an independent prognosis for patients with rectal cancer. We constructed a new prognostic nomogram that combines risk score, gender, and age as a model for predicting OS in patients with rectal cancer for clinical promotion.

The difference analysis of Hallmark pathway between HRisk and LRisk showed that it was mainly concentrated in hypoxia, metastasis, and inflammation-related pathways. Hypoxia is involved in cancer progression and can promote angiogenesis, stem cell phenotype, and malignant progression of various cancer types. Hypoxia-inducible factor-1 (HIF-1) is induced under hypoxia, increasing the transcription of downstream factors. Many studies believe that HIF-1 can cause the occurrence of Epithelial-Mesenchymal Transition (EMT), increase protein levels of EMT-associated transcription factors and mesenchymal biomarkers (vimentin, cadherin 2), and downregulate the expression of epithelial characteristics (cadherin 1) (21). In addition, the PI3K-Akt pathway is the main signal pathway to stabilize the expression of HIF-1 (22, 23). Interestingly, KEGG analysis showed that the PI3K-Akt pathway was significantly enriched. The HIF target gene is also closely related to the inflammatory response, so hypoxia may further promote tumor metastasis by regulating inflammation. Hypoxia-induced EMT in hepatocellular carcinoma leads to the increase in CCL20 secretion by hepatocellular carcinoma cells, which induces the co-cultured macrophages to express indoleamine 2,3-dioxygenase (IDO). IDO expressing cells inhibit the proliferation of T cells and promote the proliferation of immunosuppressive regulatory T cells (24). In addition to macrophages, hypoxia also regulates neutrophils. Hypoxia in the mouse colon cancer model exacerbated the formation of neutrophil extracellular traps (NETs). NET is released after surgical stress and is associated with reduced disease-free survival in patients with metastatic colorectal cancer (25). Interestingly, GO analysis found that neutrophil activation-related pathways were significantly enriched. According to the review, we found that hypoxia can directly promote metastasis and further promote metastasis by regulating inflammation.

In the tumor tissue microenvironment, there are a variety of cell mixtures, such as tumor cells, immune cells, and stromal cells (26). It is reported that tumor cells can control the microenvironment. That is, malignant glioma recruits a large number of surrounding cells and conquers them to form a protective barrier (27). Consequently, low tumor purity and associated cellular heterogeneity are the main factors leading to malignant tumor progression. This seems to explain why most treatment strategies that target glioma cells alone fail to achieve good results. Interestingly, our results also showed that the tumor purity was lower in the HRisk group with a poor prognosis. Stromal cells and immune cells constitute the main non-tumor components in the glioma microenvironment. In addition, there was a close relationship between stromal and immune scores. Gliomas with different purity mainly show different local immune states. The local immune status of low-purity gliomas is significantly enhanced (26, 28). This is consistent with our results that HRisk patients with lower tumor purity have higher stromal and immune scores. It is reported that low-purity gliomas are rich in neutrophil infiltration, which is positively correlated with grading progress, malignancy, and drug resistance (29, 30). Neutrophils and T cells have complex interactions (31). This interaction is essential for maintaining the immunosuppressive phenotype. Interestingly, by calculating the difference in immune cell infiltration between HRisk and LRisk, we found that HRisk patients had high expression of neutrophils and low expression of activated memory CD4 + T cells. It is reported that neutrophils are part of the myeloid and stromal cell network, expressing VISTA, PDL1, and CD86, driving immune checkpoints (PSGL1, PD1, and CTLA4) to inhibit the immune activity of T cells (32–36). To further explore the relationship between neutrophils and activated memory CD4 + T cells, we detected a group of lymphocyte checkpoint ligands (VISTA, PDL1, CD86) expressed by neutrophils and found that they were significantly overexpressed in HRisk patients, while the receptors expressed by T cells were also highly expressed in varying degrees. In conclusion, we found that hypoxia resulted in lower tumor purity and higher immune and interstitial scores; Hypoxia mainly leads to the enrichment of neutrophils, which further leads to the immunosuppression of memory CD + T cells. In addition, some immune checkpoints highly expressed in the HRisk group enhance the possibility of enhanced immunotherapeutic response. Therefore, it is suggested to adopt a comprehensive treatment strategy of blocking neutrophil infiltration. The peripheral blood neutrophil/lymphocyte ratio is closely related to the degree of local neutrophil infiltration, which may be an invasive index to evaluate the treatment response (37, 38).

Five hypoxia-related genes in the GEO cohort constitute prognostic markers (ANLN, Flt1, ANGPTL4, NR3C1, and SRPX). The actin-binding protein encoded by the ANLN (Anillin actin-binding protein) gene regulates cell growth, migration, and cytokines. Previous studies have shown that the high expression of ANLN predicts poor prognosis in renal cell carcinoma, liver cancer, and lung cancer (39–41), but no one has reported its role in rectal cancer. Our study found that ANLN was almost not expressed in neutrophils and low expressed in cancer tissues, which was related to the good prognosis of rectal cancer. More importantly, it is low expressed in HRisk and positively correlated with activated memory CD4 + T cells. ANLN may act as a tumor suppressor in hypoxia-mediated progression of rectal cancer. SRPX is a protein coding gene and may be involved in phagocytosis during disk shedding, cell adhesion to cells other than the pigment epithelium or signal transduction. Some studies have shown that SRPX is related to the short OS time of endometrial cancer (42), and can regulate tumor-related fibroblasts and promote the invasiveness of ovarian cancer. At present, no one has reported the role of SRPX in rectal cancer. Our study found that SRPX is almost not expressed in neutrophils and highly expressed in HRisk, which is associated with the poor prognosis of rectal cancer. More importantly, it is positively correlated with neutrophils and negatively correlated with activated memory CD4 + T cells. SRPX may play the role of cancer-promoting factor in hypoxia-mediated progression of rectal cancer, promote neutrophils, and inhibit the immune function of T cells. Angiopoietin-like 4 (ANGPTL4) is a secretory glycoprotein that regulates metastasis in tumors. It is reported that ANGPTL4-mediated glycolysis promotes the progression of colorectal cancer (43); it may promote metastasis and inhibit the apoptosis of colorectal cancer cells by upregulating BMP7 (44). In this study, ANGPTL4 was significantly overexpressed in neutrophils and was associated with a poor prognosis of rectal cancer. More importantly, it is highly expressed in HRisk, positively correlated with neutrophils, and negatively correlated with activated memory CD4 + T cells. ANGPTL4 can reflect neutrophil-mediated T cell immunosuppression to a certain extent.

## Conclusion

In conclusion, our study found new prognostic markers for five hypoxia-related genes in rectal cancer. This model has been proved to be effective in predicting OS in training and validation queues, and provides better stratification for future trials. The relationship between hypoxia-related characteristics and tumor-immune microenvironment suggests the possibility of immunotherapy in specific populations. ANLN, which plays a role in inhibiting cancer, and SRPX, which plays a role in promoting cancer, can be used as effective biomarkers to predict the prognosis and the efficacy of immunotherapy. However, the underlying mechanism is still largely unclear and needs further exploration.

## Data Availability Statement

The original contributions presented in the study are included in the article/Supplementary Material, further inquiries can be directed to the corresponding author.

## Ethics Statement

Written informed consent was obtained from the individual(s) for the publication of any potentially identifiable images or data included in this article.

## Author Contributions

KY and KG performed the data analysis and wrote the manuscript. ZS, NY, JQ, and MW contributed to the manuscript revision. ZS, NY, and JQ contributed to the literature search and data extraction. MW and KG conceived and designed the study. All authors have read and approved the final version of the manuscript.

## Conflict of Interest

The authors declare that the research was conducted in the absence of any commercial or financial relationships that could be construed as a potential conflict of interest.

## Publisher's Note

All claims expressed in this article are solely those of the authors and do not necessarily represent those of their affiliated organizations, or those of the publisher, the editors and the reviewers. Any product that may be evaluated in this article, or claim that may be made by its manufacturer, is not guaranteed or endorsed by the publisher.

## Supplementary Material

Supplementary Figure S1 Validation of Nomogram model in TCGA cohort. **(A)** Kaplan–Meier curves for the OS of patients between the high-risk and low-risk groups. **(B)** Plots depict the calibration of nomograms. **(C)** Area under the curve of time-dependent receiver operating characteristic curves. **(D–F)** Decision curve analyses of the nomograms based on OS for 1-, 3-, and 5-year risk.

Supplementary Figure S2 Cluster analysis of 342 hypoxia-related genes. **(A)** Consensus matrix heatmap based on 342 hypoxia-related genes. **(B)** Cumulative Distribution Function (CDF) was used to determine the optimal number of clusters k. **(C)** Relative change in area under the CDF curve was used to determine the optimal number of clusters k. **(D)** Kaplan–Meier curves for the overall survival of patients between cluster 2 and cluster 3 group.

Supplementary Figure S3 Potential functional analyses in the TCGA cohort. **(A)** GSVA analysis of hallmark pathways between the HRisk and LRisk groups was performed. **(B,C)** KEGG and GO pathway analyses of differentially expressed genes between the HRisk and LRisk group.

Supplementary Figure S4 The relationship between hypoxia and tumor-immune microenvironment in the TCGA cohort. Comparison of TumorPurity **(A)**, ImmuneScore **(B)**, and StromalScore **(C)** between the HRisk and LRisk patients. **(D)** Boxplots depicting the CIBERSORT scores of 22 immune cells of the HRisk patients compared to the LRisk patients. **(E)** Boxplots depicting the Xcell scores of 64 immune cells of the HRisk patients compared to the LRisk patients.

Supplementary Table S1 Training set GSE87211.

Supplementary Table S2 Validation set TCGA.

Supplementary Table S3 342 Hypoxia-related genes and 79 Immune checkpoint.

Supplementary Table S4 Clinical characteristics of the rectal cancer patients used in this study.

Supplementary Table S5 Baseline characteristics of the patients in different risk groups.
