# Supplementary figures and images for: Development and Validation of a Novel Hypoxia Score for Predicting Prognosis and Immune Microenvironment in Rectal Cancer
Source: Front Surg. 2022 Apr 25;9:881554. doi: 10.3389/fsurg.2022.881554 (PMC9081503; doi:10.3389/fsurg.2022.881554)

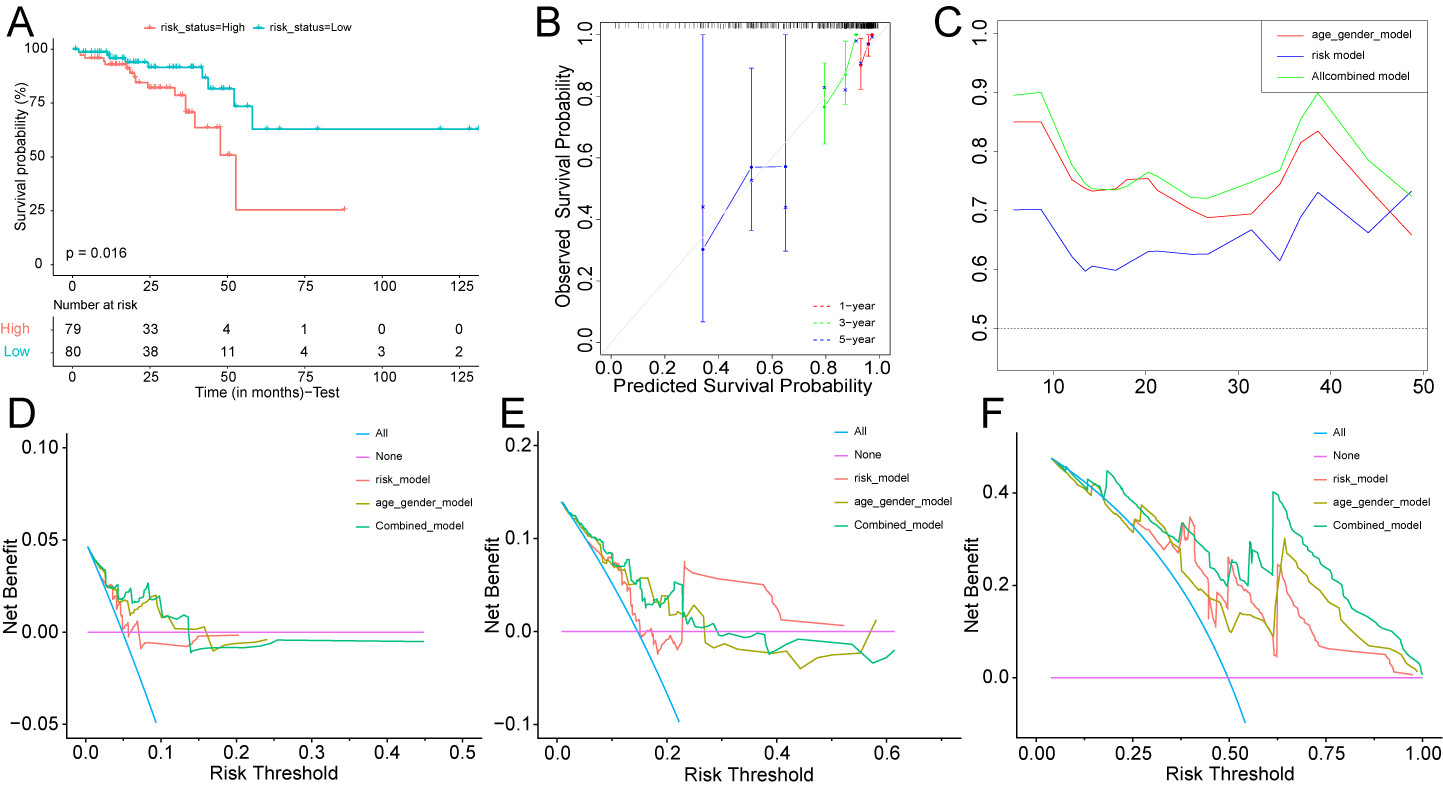

Supplement: Supplementary Figure S1 — Validation of Nomogram model in TCGA cohort. (A) Kaplan–Meier curves for the OS of patients between the high-risk and low-risk groups. (B) Plots depict the calibration of nomograms. (C) Area under the curve of time-dependent receiver operating characteristic curves. (D–F) Decision curve analyses of the nomograms based on OS for 1-, 3-, and 5-year risk. [file Image_1.TIF]

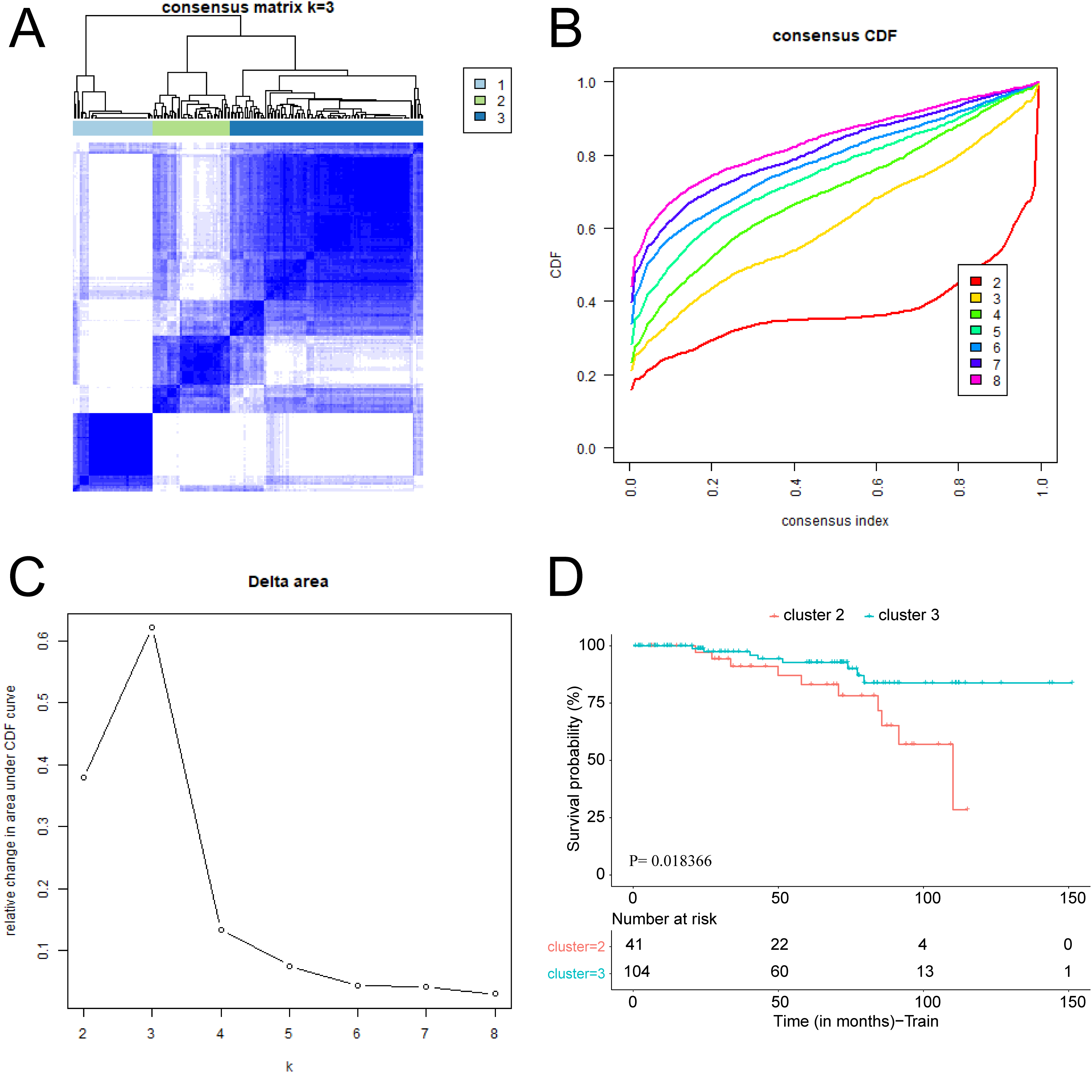

Supplement: Supplementary Figure S2 — Cluster analysis of 342 hypoxia-related genes. (A) Consensus matrix heatmap based on 342 hypoxia-related genes. (B) Cumulative Distribution Function (CDF) was used to determine the optimal number of clusters k. (C) Relative change in area under the CDF curve was used to determine the optimal number of clusters k. (D) Kaplan–Meier curves for the overall survival of patients between cluster 2 and cluster 3 group. [file Image_2.TIF]

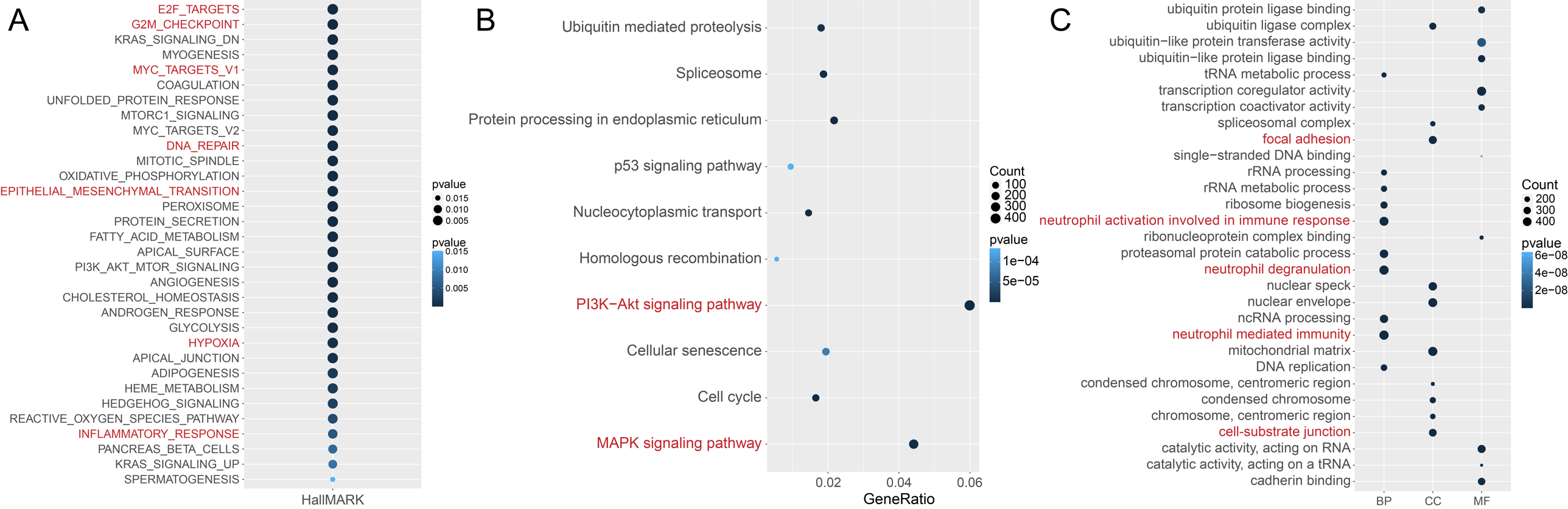

Supplement: Supplementary Figure S3 — Potential functional analyses in the TCGA cohort. (A) GSVA analysis of hallmark pathways between the HRisk and LRisk groups was performed. (B,C) KEGG and GO pathway analyses of differentially expressed genes between the HRisk and LRisk group. [file Image_3.TIF]

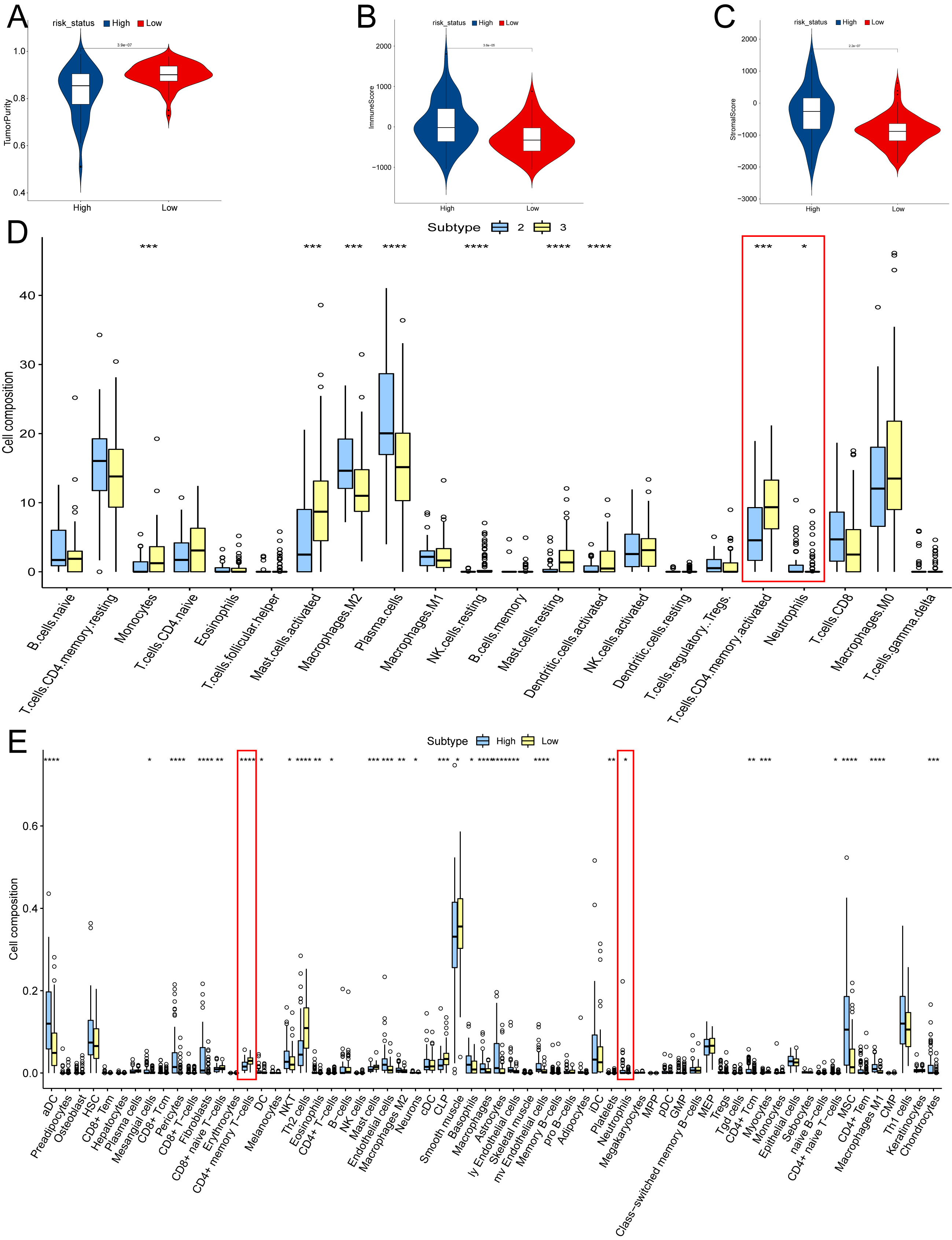

Supplement: Supplementary Figure S4 — The relationship between hypoxia and tumor-immune microenvironment in the TCGA cohort. Comparison of TumorPurity (A), ImmuneScore (B), and StromalScore (C) between the HRisk and LRisk patients. (D) Boxplots depicting the CIBERSORT scores of 22 immune cells of the HRisk patients compared to the LRisk patients. (E) Boxplots depicting the Xcell scores of 64 immune cells of the HRisk patients compared to the LRisk patients. [file Image_4.TIF]
