# Supplementary material for: Development and Validation of a Novel Hypoxia Score for Predicting Prognosis and Immune Microenvironment in Rectal Cancer
Source: Front Surg. 2022 Apr 25;9:881554. doi: 10.3389/fsurg.2022.881554 (PMC9081503; doi:10.3389/fsurg.2022.881554)
Supplement: Supplementary Table S4 — Clinical characteristics of the rectal cancer patients used in this study. [file Table_4.DOCX]

| **Supplementary Table S4. Clinical characteristics of the rectal cancer patients used in this study.** | | |
| --- | --- | --- |
|  | GEO cohort (n = 190) | TCGA cohort (n = 159) |
| **Age (%)** |  |  |
| <60 | 70 (36.8) | 49 (30.8) |
| ≥60 | 120 (63.2) | 110 (69.2) |
| **Gender (%)** |  |  |
| Female | 58 (30.5) | 71 (44.7) |
| Male | 132 (69.5) | 88 (55.3) |
| **OS. Days (Mean ± SD)** | 1722 ± 1075 | 807 ± 642 |
