# Supplementary material for: Development and Validation of a Novel Hypoxia Score for Predicting Prognosis and Immune Microenvironment in Rectal Cancer
Source: Front Surg. 2022 Apr 25;9:881554. doi: 10.3389/fsurg.2022.881554 (PMC9081503; doi:10.3389/fsurg.2022.881554)
Supplement: Supplementary Table S5 — Baseline characteristics of the patients in different risk groups. [file Table_5.DOCX]

| **Supplementary Table S5. Baseline characteristics of the patients in different risk groups.** | | | | | | |
| --- | --- | --- | --- | --- | --- | --- |
|  | GEO cohort | | pvalue | TCGA cohort | | pvalue |
|  | HRisk  (n = 95) | LRisk  (n = 95) |  | HRisk  (n = 79) | LRisk  (80) |  |
| **Age (%)** |  |  | 0.367 |  |  | 0.25 |
| <60 | 38 (40) | 32 (33.7) |  | 21 (26.6) | 28 (35) |  |
| ≥60 | 57 (60) | 63 (66.3) |  | 58 (73.4) | 52 (65) |  |
| **Gender (%)** |  |  | 0.059 |  |  | 0.93 |
| Female | 35 (36.8) | 23 (24.2) |  | 35 (44.3) | 36 (45) |  |
| Male | 60 (63.2) | 72 (75.8) |  | 44 (55.7) | 44 (55) |  |
| **OS. Days (Mean ± SD)** | 1570 ± 1011 | 1873 ± 1127 | 0.053 | 706 ± 473 | 906 ± 760 | 0.049 |
